# Lachesin is a mosquito receptor for multiple arthritogenic alphaviruses

**DOI:** 10.64898/2026.07.28.741058

**Published:** 2026-07-28

**Authors:** Jesse S. Plung, Enzo Mameli, Wanyu Li, Anja C. M. de Bruin, Jessica A. Plante, Xiaoyi Fan, Biswajit Das, Bailey C. Willett, Yanhui Hu, Ellie M. Hajovsky, Haley Varnum, Praju V. Anekal, Xiaomei Sun, Kate Thornburg, Vesna Brusic, Catherine E. Hammond, Paula Montero Llopis, Raghuvir Viswanatha, W. Robert Shaw, Flaminia Catteruccia, Scott C. Weaver, Kenneth S. Plante, Gisa Gerold, Norbert Perrimon, Jonathan Abraham

## Abstract

Arthritogenic alphaviruses cause acute febrile illnesses associated with rash and arthritis when they are transmitted to humans through the bite of infected mosquitoes. Among these, chikungunya virus (CHIKV), transmitted primarily through the bite of infected *Aedes aegypti* and *Aedes albopictus* mosquitoes, causes explosive outbreaks involving hundreds of thousands to millions of cases, with recent re-emergence in several global regions. While several cellular receptors that mediate alphavirus entry into mammalian cells have been identified, their mosquito counterparts remained unknown, largely due to a lack of functional genomics tools for these invertebrate species. Here, we established a CRISPR-based genetic screening platform in *Aedes albopictus* cells and used it to identify Lachesin, a conserved invertebrate cell adhesion molecule, as a receptor for CHIKV and multiple related alphaviruses including Semliki Forest virus (SFV), o’nyong-nyong virus (ONNV), Mayaro virus (MAYV), and Ross River virus (RRV). Lachesin depletion using RNA interference, anti-Lachesin antibody treatment, and soluble forms of Lachesin blocked CHIKV and SFV E2–E1 glycoprotein-mediated infection of mosquito cells. We show that alphavirus E2–E1 glycoproteins bind the first immunoglobulin domain of Lachesin, facilitating attachment and internalization of virus-like particles. Orthologs from divergent mosquito genera, but not from arachnids or other arthropods, also serve as alphavirus receptors, suggesting that cellular receptor binding is not the main obstacle to arthritogenic alphavirus vector host expansion. Our findings enhance understanding of the mechanisms of alphavirus emergence and vector competence and could aid in the development of broadly active, entry-targeted therapeutics against multiple alphaviruses that threaten public health.

## Full Text Availability

The license terms selected by the author(s) for this preprint version do not permit archiving in PMC. The full text is available from the preprint server.

